# Alloying and Processing Effects on the Microstructure, Mechanical Properties, and Degradation Behavior of Extruded Magnesium Alloys Containing Calcium, Cerium, or Silver

**DOI:** 10.3390/ma13020391

**Published:** 2020-01-15

**Authors:** Jan Bohlen, Sebastian Meyer, Björn Wiese, Bérengère J. C. Luthringer-Feyerabend, Regine Willumeit-Römer, Dietmar Letzig

**Affiliations:** 1 Magnesium Innovation Centre (MagIC), Helmholtz-Zentrum Geesthacht, Max-Planck-Str. 1, D21502 Geesthacht, Germany; sebastian.meyer@hzg.de (S.M.); dietmar.letzig@hzg.de (D.L.); 2 Metallic Biomaterials, Helmholtz-Zentrum Geesthacht, Max-Planck-Str. 1, D21502 Geesthacht, Germany; bjoern.wiese@hzg.de (B.W.); berengere.luthringer@hzg.de (B.J.C.L.-F.); regine.willumeit@hzg.de (R.W.-R.)

**Keywords:** magnesium alloys, zinc, calcium, silver, rare earth, extrusion, microstructure, mechanical properties, degradation, cytocompatibility

## Abstract

Magnesium alloys attract attention as degradable implant materials due to their adjustable corrosion properties and biocompatibility. In the last few decades, especially wrought magnesium alloys with enhanced mechanical properties have been developed, with the main aim of increasing ductility and formability. Alloying and processing studies allowed demonstrating the relationship between the processing and the microstructure development for many new magnesium alloys. Based on this experience, magnesium alloy compositions need adjustment to elements improving mechanical properties while being suitable for biomaterial applications. In this work, magnesium alloys from two Mg-Zn series with Ce (ZE) or Ca (ZX) as additional elements and a series of alloys with Ag and Ca (QX) as alloying elements are suggested. The microstructure development was studied after the extrusion of round bars with varied processing parameters and was related to the mechanical properties and the degradation behavior of the alloys. Grain refinement and texture weakening mechanisms could be improved based on the alloy composition for enhancing the mechanical properties. Degradation rates largely depended on the nature of second phase particles rather than on the grain size, but remained suitable for biological applications. Furthermore, all alloy compositions exhibited promising cytocompatibility.

## 1. Introduction

A high potential for the application of wrought magnesium alloys has been directed to applications in structural lightweight construction, but increasingly to their suitability as a body compatible implant materials [1,2]. While the development of magnesium alloys for structural applications focusses on corrosion protection, i.e., corrosion tolerant alloys, as well as surface protection, the tendency to degradation is a desired property for non-permanent implant applications. Compared to permanent implants, bioresorbable implants allow avoiding second or revision surgery [3]. Furthermore, the bioactive properties of magnesium, which support regenerative processes, have been shown. The osteoconductivity and osteoinductivity of magnesium implants could be proven in vivo, resulting in accelerated and sustainable bone fracture healing [4,5]. In comparison to biocompatible polymers or other metals, magnesium based materials possess mechanical properties more similar to those of bones [2].

For biomedical applications, the requirements of the applied alloys differ from those needed for structural applications. As a prerequisite, the selection of alloying elements for a property design is limited to non-toxic, biologically available ones, allowing controlled degradation rates in the physiological environment. Although very different reactivities of the materials are required in biomedical and structural application environments, both areas of application have high and similar demands on the homogeneity of the semi-finished products, their mechanical properties, and their formability. Furthermore, alloying windows may vary significantly if economic questions are considered.

In this regard, a preliminary understanding of the impact of single alloying elements on magnesium materials properties can be summarized. Zinc (Zn) is known to increase the mechanical strength properties, as well as the ductility of the magnesium alloys [6,7]. A higher tolerance limit for nickel, copper, and iron impurities and a lower corrosion rate gradient above the tolerance limit were also found [8,9]. Zn is also known as an essential element for the body [10]. Zirconium (Zr) is known as a powerful grain refiner in magnesium alloys. As such, it cannot be used in combination with aluminum or silicon in the melt during casting [11]. Rare earth elements and magnesium show a similar potential in the galvanic series of elements. Thus, the neutral behavior of rare earth containing magnesium alloys is expected. In recent works, this neutral behavior has been confirmed for gadolinium (Gd) as a sole alloying element to magnesium [12]. Gd, even if remaining a question of debate, is an interesting element for musculoskeletal application, as it was shown to support hydroxyapatite formation by osteoblasts in vitro [13,14] and suitable bone healing in vivo [15,16]. Cerium (Ce) is used in magnesium to form a fine grained microstructure and relatively weak textures during extrusion [17]. However, praseodymium, cerium, or yttrium has been shown to deposit in the liver and the bone, where they can lead to hepatosis if concentrations are high [18]. Still, WE43 is a yttrium and rare earth (Nd-mischmetal) containing magnesium alloy applied in human implants such as stents [19], screws, pins, or fixations [20]. Silver (Ag) could also be an interesting alloying element for biomedical applications according to its antibacterial properties. A significantly lower survival rate of the pathogenic bacteria *Staphylococcus aureus* (DSMZ 20231) and *Staphylococcus epidermidis* (DSMZ 3269) has been demonstrated [21]. In addition, the corrosion rate increased strongly with increasing silver content if the element was not kept in solid solution [22]. However, reasonable corrosion resistance and cytocompatibility can be achieved and have been reported from immersion tests with osteoblastic cell lines [21,23]. Calcium (Ca) shows a strong influence on the texture development during rolling or extrusion [24,25], especially leading to weaker textures without a strong alignment of basal planes. Such textures are known to enable improved ductility and also lower anisotropic mechanical behavior [26]. As the most abundant mineral in the body, Ca is predominantly found in bone, and the release of Ca ions regulates the activation of osteoblasts and osteoclasts to facilitate bone regeneration in vitro [27], but also in vivo [28].

Besides alloy composition, the respective properties (especially mechanical and corrosion properties) are distinctly determined by the applied processing and the concurrent microstructure development. Extrusion, as a massive forming process, includes a high activity of deformation mechanisms, as well as dynamic recrystallization because it needs to be carried out at elevated temperatures [29]. Temperature and deformation rate influence the microstructure development distinctly, which also changes the related mechanical properties. The results often confirm distinct anisotropy of the mechanical properties, especially if tensile and compressive stresses are applied.

In this context, the effect of dilute alloys with a low amount of the above suggested alloying elements is considered in this work for the extrusion of round bars with recrystallized microstructures from varied extrusion parameter settings. In a series of alloys with Zn and Ce, a higher Zn containing counterpart to a classical ZE10 [30] has been suggested with varied content of pure Ce (instead of non-pure cerium based mischmetal), alloys ZE10, ZE21, and ZE21+. In another series of alloys, a more dilute version of a Ca containing counterpart of a ZE10 alloy has been investigated, in one example also containing Zr as grains a refiner, ZX00 and ZXK000 [31]. A further series of alloys is based on a rather dilute Mg-Ag alloy QX20 [22] with Ca as an additional alloying element as a texture modifier, as well as Zr as a grain refiner, QXK100 and QXK200. The investigations stretch from microstructure and texture characterization and their relation to the mechanical properties, as well as the influence of the microstructures on the degradation behavior revealed from degradation tests under physiological conditions and the cytocompatibility observed via live/dead stainings of cells directly cultured on various material surfaces.

## 2. Materials and Methods

### 2.1. Casting and Extrusion

The magnesium alloy composition of three series was composed from pure elements Mg (high purity ingots, 99.98%), Ce, Ca, and Ag. A binary Mg-Zr master alloy Zirmax (Mg-33%Zr) was added to enable the addition of Zr as a grain refiner. The alloys were molten at 750 °C using sulfur hexafluoride and argon as protective atmosphere and stirred (ca. 250 rpm) for a homogeneous distribution of the alloying elements. A modified gravity casting process into crucibles and directional solidification of the melt in a water bath were applied. The cast materials were machined into billets for extrusion with a diameter of 93 mm to fit a container with a diameter of 95 mm. The billet length was 150 mm. The alloy labels (corresponding to ASTM descriptions) and the composition revealed by optical emission spectroscopy and X-ray fluorescence analysis are collected in Table 1. A homogenization heat treatment was carried out for 16 h at 450 °C prior to extrusion.

The extrusion of round bars with a diameter of 17 mm (extrusion ratio 1:31) was carried out using an 8 MN extrusion press (Extrusion Research & Development Centre, TU Berlin, Germany). The used die was modified with a drill hole to place a thermocouple close to the inner surface for a measurement of the resulting temperature close to the forming zone. Extrusion data therefore included the required extrusion force, as well as the resulting forming temperature, which differed from the billet temperature due to deformation heating. Extrusion temperatures varied between 250 °C and 300 °C, and extrusion speeds varied from 1 to 5 mm/s, respectively. Only for the QX-series alloys QX20, QXK100, and QXK200, the speed remained lower at 3 mm/s in order to avoid hot cracking.

### 2.2. Characterization

Standard metallography procedures of sample preparation included picric acid as an etchant [32] to reveal grains and grain boundaries in light optical microscopy (Leica DMI 5000M). Polished surfaces of longitudinal sections of the samples were also used for texture measurements by X-ray diffraction with an applied goniometer system (Panalytical X’Pert Pro). Six reflections were measured to pole figures up to a tilt angle of 70° for a recalculation of the orientation distribution function and presentation of inverse pole figures parallel to the extrusion direction (ED). An open source toolbox MTEX was applied for this purpose [33]. Uniaxial tension and compression tests parallel to the ED were carried out using a constant initial strain rate of 10^−3^ s^−1^ at ambient temperature on a universal testing machine (Zwick Z050). Dog bone samples with screws and a diameter of 6 mm were machined for tension tests, as well as cylindrical samples with a diameter of 11 mm and a length of 17 mm for compression tests. A detailed analysis of the chemistry of precipitates was not carried out in this work, but a limited view of the impact of alloy specific precipitates was chosen. However, from a thermodynamic database consideration (Pandat^TM^), different types of stable precipitates could be described: The ZX-series alloys would maintain the alloying elements in solid solution above 425 °C, but form a stable Mg_2_Ca phase below this temperature [34]. The small addition of Zr could interact with Zn. No solid solution temperature range was available for the ZE-series alloys, but a stable second phase of Mg_12_Ce. The QX-series alloys did not exhibit known interaction between Ca and Ag, but led to the formation of the stable the Mg_2_Ca and Mg_4_Ag phases. However, in earlier work, another chemistry of Mg-Ag intermetallics was suggested, e.g., Mg_54_Ag_17_ [22]. The addition of Zr seemed to remain in solid solution.

### 2.3. Degradation and Cytocompatibility Tests

Discs with a diameter of 10 mm and 2 mm thickness were machined from the extruded bars. The discs sides were ground at low speed with increasing SiC paper grits (1200, 2500, and 4000) and rinsed with ethanol (Sigma-Aldrich Chemie GmbH, Munich, Germany). The discs were then cleaned by sonication for 20 min in n-hexane, acetone, and undiluted ethanol and finally sterilized in 70% ethanol (all chemicals were purchased from Sigma-Aldrich Chemie GmbH, Munich, Germany). Dried samples were stored sterile under vacuum until use.

For the degradation assays, 3–6 samples were weighted (W1) and placed in 24 well plates, and 3 mL of α-minimum essential medium (MEM; Life technologies, Darmstadt, Germany) supplemented with 10% fetal bovine serum (FBS; Fisher Scientific GmbH, Schwerte, Germany) and 10 µL/mL penicillin-streptomycin were added. After 72 h incubation under physiological conditions (5% CO_2_, 20% O_2_, 95% humidity, 37 °C), samples were treated with chromic acid (Sigma-Aldrich Chemie GmbH, Munich, Germany) twice for 10 min for the removal of the degradation layer and weighed again (W2). The degradation rate (*DR*) was determined following the ASTM Norm G31-72 [35] as:

$$DR=\frac{\Delta m}{A\cdot t\cdot p}$$

where Δ*m* (W1−W2) represents the weight loss, *A* the surface area, *t* the immersion time, and *ρ* the sample density (measured using an Archimedean balance). Thus, the degradation rate was obtained as a decomposition depth of the sample for a given period, here recalculated for a year.

Cytocompatibility was assessed via live/dead staining of human umbilical cord perivascular cells (HUCPV) directly cultured on three samples. HUCPV cells are mesenchymal stem cells (MSC) with a high proliferation rate and strong potential for differentiation into the skeletal lineages (both bone and cartilage) [36,37]. HUCPV were isolated from the vessels’ surface of umbilical cords, (acquired from consenting donors; Perinatalzentrum Asklepios Klinikum Altona, Hamburg, Germany). This isolation was performed according to the protocols of Sarugaser et al. [37] and approved by the Ethics Committee of the Hamburg Medical Association (Hamburg, Germany). Here, three samples were preincubated overnight in cell culture medium (the same as for the degradation experiment, i.e., α-MEM) to avoid initial degradation burst. Afterwards, 5000 cells were seeded onto the material surface (in beforehand agarose coated wells, 12 well plate), allowed to adhere for 30 min, and then, 2 mL fresh medium were added. Cells were further cultured for 7 days, and then, staining was performed. Therefore, the medium was exchanged with fresh medium supplemented with calcein-AM (2 µM; green), ethidium homodimer I (5 µM; red), and Höchst 33342 (1 µg/mL; blue for the cell nucleus). The live cells (green) and the dead cells (red) were visualized using fluorescence microscopy and NIS-Elements Microscope Imaging Software 3.2 (Nikon GmbH, Dusseldorf, Germany).

## 3. Results

### 3.1. Extrusion Experiments

Table 2 collects the results of the extrusion forces and measured die temperatures from steady state flow sections during the extrusion experiments. During slow extrusion at 250 °C, no stable flow was realized for the Mg-Zn-Ca- and the Mg-Ag-Ca- series alloys. In such cases, a force build-up occurred, which was then released with a force drop and a concurrent sequential increase of the extrusion speed. This behavior resulted in an unstable flow in the form of an oscillation of the force and the speed, respectively. Only at higher extrusion speed, such a behavior could be avoided. Then, steady state forces decreased with increasing billet temperature, but also with the increase of the extrusion speed. The temperatures measured in the die (associated with the temperature in the forming region of the profile) showed a significant increase from the initial billet temperature, which was also higher at higher extrusion speed. Interestingly, except for ZX00, after fast extrusion, the extrusion temperature remained lower or comparable at the higher initial billet temperature.

For the ZE-series alloys, there was an increase of the extrusion force with increasing Ce content. Again, there was a decrease of the extrusion force with increasing billet temperature, but also with increasing extrusion speed in some cases.

### 3.2. Characterization of Mg-Zn-Ca (Zr) Alloys: ZX-Series

**Figure 1:**
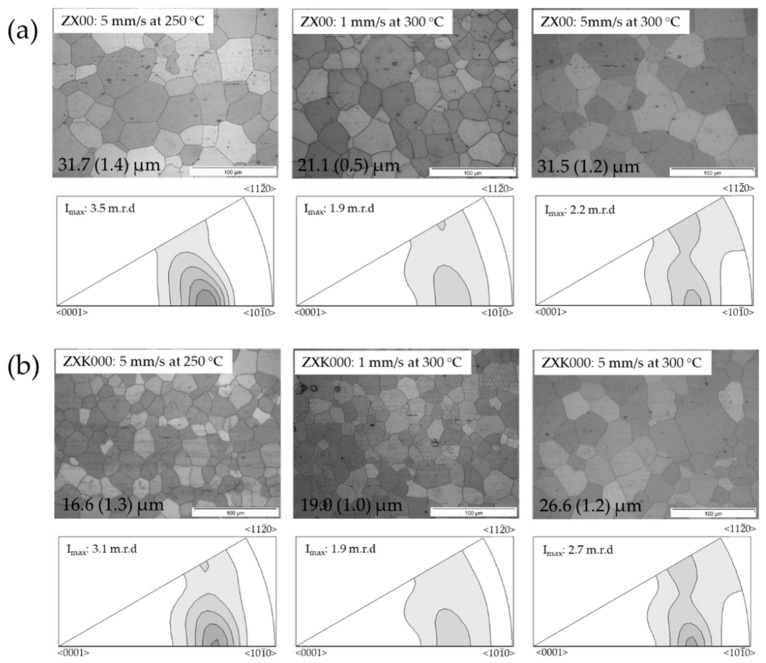
Micrographs from longitudinal sections (extrusion direction (ED) horizontal) and inverse pole figures in the extrusion direction of extruded Mg-Zn-Ca alloy (ZX00) at varied temperatures and extrusion speeds: (**a**) ZX00; (**b**) ZXK000.

For ZX00 and ZXK000, micrographs from longitudinal sections of the bars after extrusion at varied temperature and speed are shown in Figure 1 together with inverse pole figures parallel to the extrusion direction (ED). Homogeneous, fully recrystallized microstructures were obtained. Some remaining particles were distributed throughout the grain structure, partly in the form of particle stringers elongated into ED (horizontal). For ZX00 (Figure 1a), the resulting grain sizes were rather large. The increase of the extrusion temperature between 250 °C and 300 °C did not have a visible effect, revealing average sizes of 32 µm in both cases. However, the extrusion speed had a visible effect on the development of the average grain size, leaving it lower at 21 µm if the speed was kept lower. The textures remained weak. A component revealing a tilt of basal planes of approximately 30° was found. There seemed to be a higher concentration with tilt out of the <10$\bar{1}$0> direction rather than the <11$\bar{2}$0> direction at the lower temperature. At higher temperature, the texture remained even weaker. For ZXK000, very similar results as for ZX00 are collected in Figure 1b. The microstructures were fully recrystallized, but the average grain sizes were smaller compared to the Zr-free alloy. In addition, the textures were very comparable. The tilt component out of the <10$\bar{1}$0> pole was stronger compared to ZX00 and especially pronounced at higher extrusion speed or low temperature. There was no visible correlation to the grain size.

**Figure 2:**
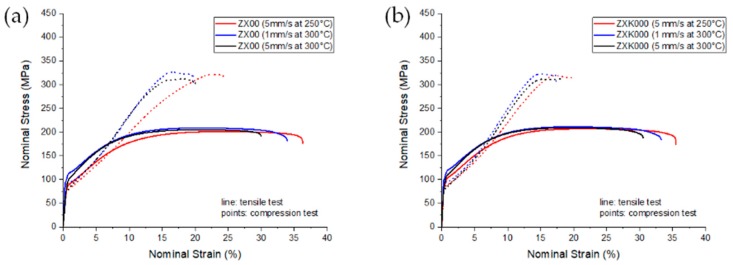
Stress–strain diagrams from tension (solid lines) and compression (dotted lines) tests at ambient temperature: (**a**) ZX00; (**b**) ZXK000.

Stress–strain diagrams of both alloys are shown in Figure 2, and the corresponding mechanical properties are collected in Table 3. A distinct difference in the yielding and strain hardening behavior between tensile and compression testing became obvious. In both alloys, ZX00 and ZXK000, S shaped flow curves during compression were found where the increase of the slope with strain was lower after extrusion at 250 °C rather than at the higher temperature, which corresponded to the strength of the texture. The stronger alignment of basal planes with a tilt out of ED corresponded to lower tensile yield stress (TYS) and compressive yield stress (CYS), but also to a lower difference between the two values, i.e., a low tension–compression yield asymmetry resulted. Furthermore, a grain size dependency was revealed. Besides the texture effect, the yield asymmetry also increased with increasing grain size. The addition of Zr to ZX00 led to a grain refinement at the same processing parameters and higher yield stresses in tension and compression. The ultimate strength (ultimate tensile stress (UTS) and ultimate compressive stress (UCS)) was not much affected with the alloying addition or with the variation of the processing parameters. The uniform and fracture strains were remarkably high for these two alloy series.

### 3.3. Characterization of Mg-Zn-Ce Alloys: ZE-Series

**Figure 3:**
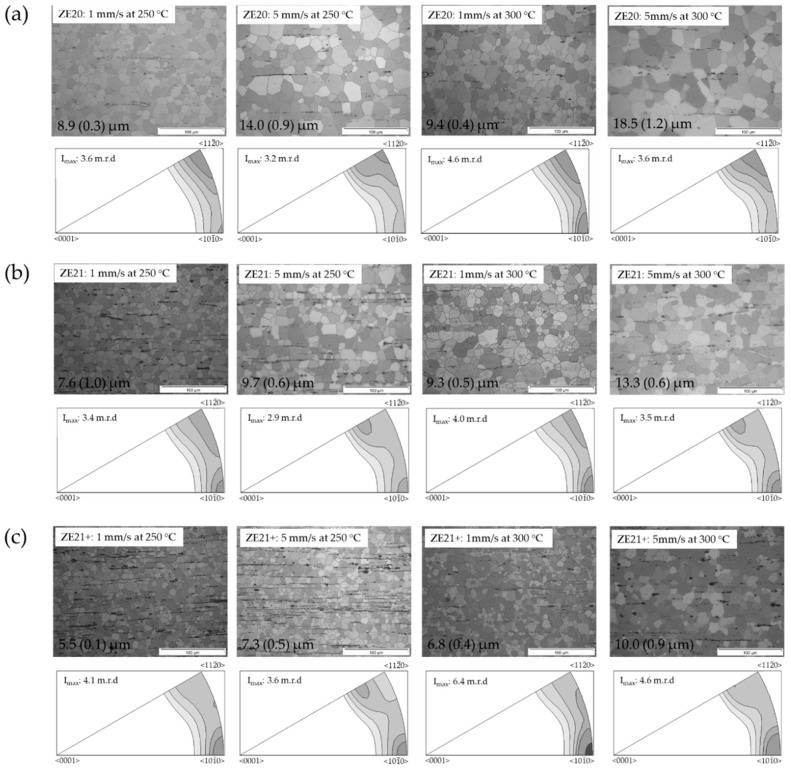
Micrographs from longitudinal sections and inverse pole figures in the extrusion direction of extruded Mg-Zn-Ce alloy (ZE-series alloys) at varied temperatures and extrusion speeds; numbers indicate average grain sizes: (**a**) ZE20; (**b**) ZE21; (**c**) ZE21+.

Figure 3 shows micrographs and related inverse pole figures of the extruded samples of the ZE-alloy series. Again, fully recrystallized microstructures were obtained. Some particles appeared preferentially as stringers parallel to the extrusion direction. The grain sizes were lower compared to the results of the ZX-series alloys at the same process parameters of extrusion. However, grain growth was obtained with both, increasing speed or temperature. The texture was more of the classical type, i.e., basal planes were aligned along ED, and the highest intensities were found at the <10$\bar{1}$0> and <11$\bar{2}$0> poles. With increasing speed, a tendency to lower intensities at the <10$\bar{1}$0> pole was found. The increase of the temperature did not lead to visible changes.

If the content of Ce increased, ZE21, the results remained comparable, but grain growth was somewhat retarded with increasing temperature or extrusion speed, with smaller grained structures obtained. The content of stringer particles also increased. For the textures, a <10$\bar{1}$0> fiber component was more visible compared to the dilute alloy ZE20. Only at higher speed and concurrent grain growth, there was the development of a component that does not lead to higher intensities at the <11$\bar{2}$0> pole, but with a tilt. The resulting orientation was around a <11$\bar{2}$1>-pole, which has often been found in rare earth containing alloys, thus labelled a “rare earth component” [38]. At lower extrusion speed, this component was not visible.

A further increase of the Ce content in alloy ZE21+ led to an enhancement of the above mentioned features of the microstructure. The grain sizes remained small, and the tendency to grain growth with speed or temperature was limited. The <10$\bar{1}$0> texture component included the highest intensities of the pole figures, and it was stronger at both higher temperature and speed. Again, the rare earth texture component was revealed in the samples after higher speed extrusion.

**Figure 4:**
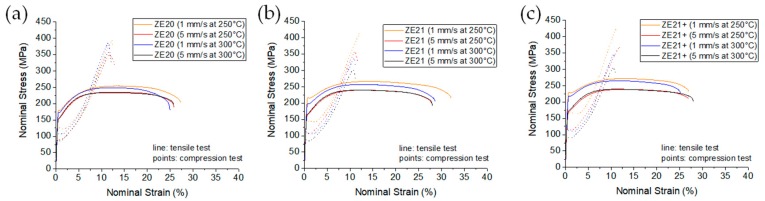
Stress–strain diagrams from tension and compression tests at ambient temperature: (**a**) ZE20; (**b**) ZE21; (**c**) ZE21+.

Figure 4 shows example stress–strain diagrams of the three ZE-alloy series with only small variations at increasing Ce levels. While the difference in the flow behavior in tension and compression was distinct again, there was no significant change with the extrusion processing parameters, temperature, and speed. Only at low temperature and low speed, a pronounced elastic limit was found in the tension test. The resulting mechanical properties, Table 4, correlated with the grain size, resulting in higher TYS and CYS with smaller grain sizes. Fracture strains were still high, but did not reach the levels of the ZX-alloy. Concurrent to the grain refinement with increasing content of Ce, there was also an incline of both the yield stresses and the strength in both tests. The yield asymmetry was distinctly higher than in the ZX-alloys, but did not vary much with the content of Ce. However, the increase of the extrusion temperature and the corresponding decrease of the yield stresses were more pronounced in compression rather than in tension.

### 3.4. Characterization of Mg-Ag-Ca (Zr) Alloys: QX-Series

**Figure 5:**
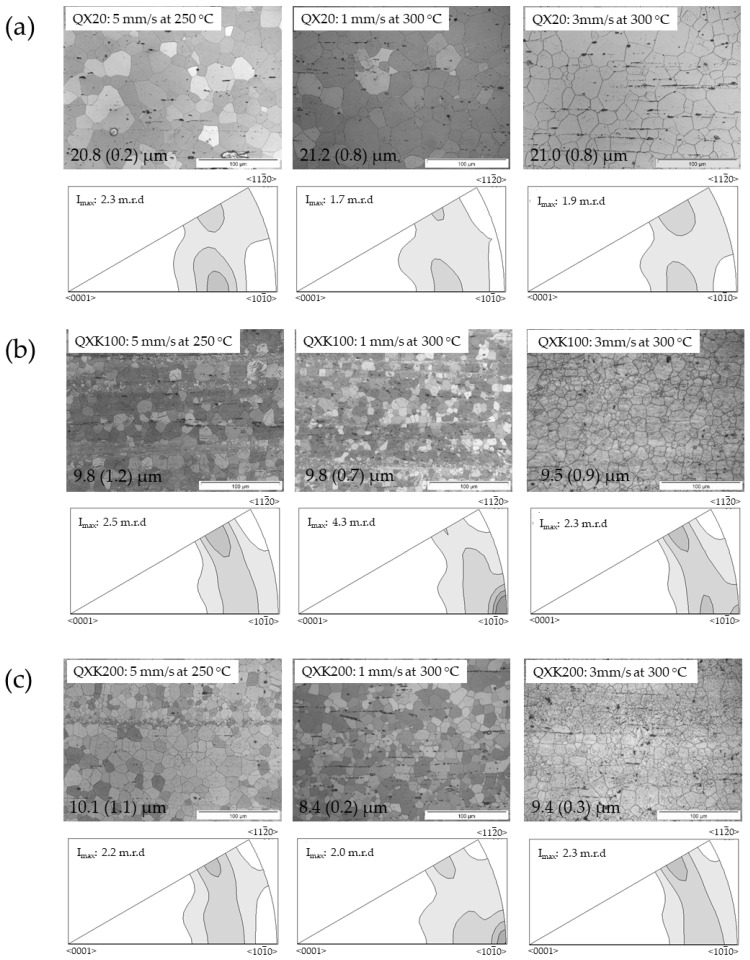
Micrographs from longitudinal sections and inverse pole figures in the extrusion direction of extruded Mg-Ag-Ca alloy (QX-series alloys) at varied temperatures and extrusion speeds; numbers indicate average grain sizes: (**a**) QX20; (**b**) QXK100; (**c**) QXK200.

The microstructures and textures of the three alloys of the QX-series alloys are shown in Figure 5. The variation of the alloy composition was the content of Ag with 1 or 2 wt.%, respectively. As the alloys contained the same amount of Ca, they were comparable to a version of the ZX-alloys with a substitution of Zn to Ag.

Like in the ZX-alloys, the effect of a Zr addition was also revealed. QX20, without Zr, did not show a variation of the microstructure with the variation of the processing parameters. An average grain size of 21 µm of the fully recrystallized microstructures was again accompanied by a distribution of particles and stringer precipitates. The texture was the same type as for ZX00, revealing a tilt of basal planes out of the ED with low strength and only slight variations in intensity. The addition of Zr in the two alloy variants QXK100 and QXK200 showed some differences, respectively, compared to QX20. However, the grain structures remained very similar with varied extrusion parameters for both alloys. Finer grained microstructures were not fully homogeneous in tendency, revealing bands of smaller grains parallel to ED. This finding of partly or inhomogeneously recrystallized microstructures vanished at high temperature and high speed. The grain sizes were very comparable in all conditions and for both alloys and visibly more finely grained compared to QX20; obviously again a grain refining effect of Zr. The textures of the fast extruded samples compared well to the basal tilt textures of QX20 with a slightly higher pronunciation of the rare earth texture component. After slow extrusion, but also after fast extrusion of QXK100 at 300 °C, this component was accompanied by a <10$\bar{1}$0> fiber component as well.

**Figure 6:**
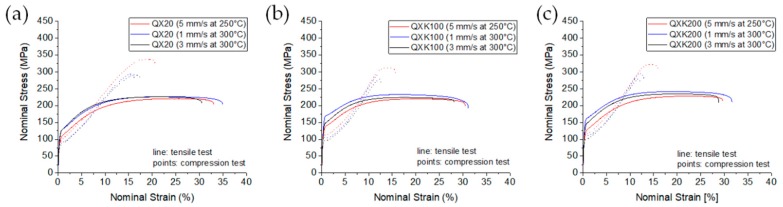
Stress–strain diagrams from tension and compression tests at ambient temperature: (**a**) QX20; (**b**) QXK100; (**c**) QXK200.

As the differences in the grain sizes for each alloy vanished, the variations in the mechanical properties (see Table 5) would rather be related to the differences in the texture of the materials. Only the grain refinement due to Zr shifted the stress levels of ZXK100 and ZXK200 (TYS, UTS, CYS) higher compared to QX20. Furthermore, the flow behavior in tension and in compression principally did not change with the alloy or the processing parameters; see Figure 6. Generally, the stress levels were slightly higher compared to ZX00 due to the more finely grained microstructure. For QX20, low yield stresses were found after extrusion at 250 °C. In this condition, the tilt component of basal planes was somewhat more pronounced compared to the extrusions at 300 °C. At the higher temperature, basically an increase of TYS was found, not CYS, thus an increase of the tension–compression yield asymmetry. For QXK100 and QXK200, this respective tilt component was even stronger, leading to low yield anisotropy. In the cases where the classical <10$\bar{1}$0> texture component was found (expressed at low speed), a higher yield anisotropy resulted mostly due to an increase of TYS. In summary, slight changes of the texture could be correlated to the visible changes in the mechanical properties.

### 3.5. Degradation and Cytocompatibility Tests

**Figure 7:**
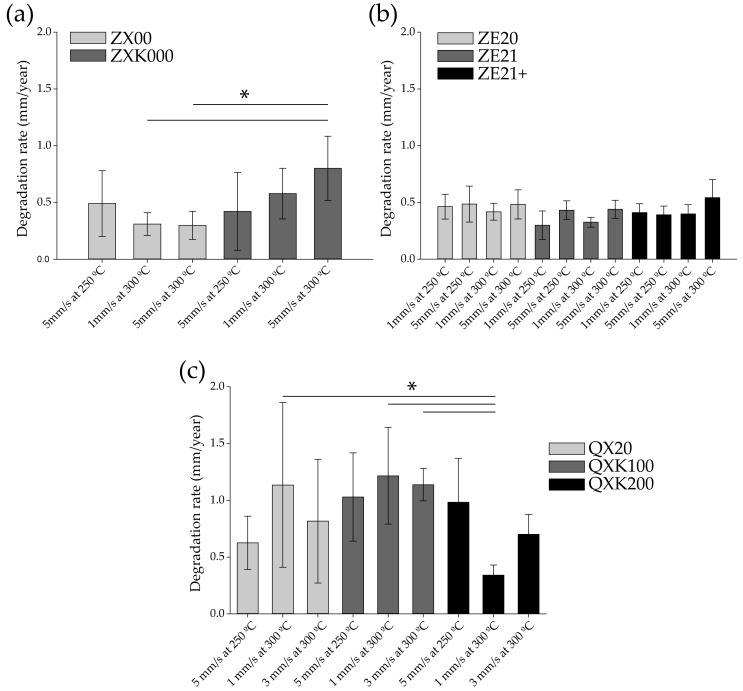
Degradation rates (mm/year) of the different alloy series: (**a**) ZX, (**b**) ZE, and (**c**) QX. Bars represent the arithmetical mean ± standard deviation (SD). Stars indicate statistically significant difference between treatments (* *p* ≤ 0.05).

Figure 7 presents the degradation rates (DR) of the studied materials. As several methods exist to determine degradation rates, cytocompatibility, and biocompatibility, a consensus about a suitable degradation rate for biological applications is difficult. However, with our methods (e.g., degradation rate under physiological conditions) and our accumulated knowledge about in vitro and in vivo experiments, we suggested that a degradation rate lower than 1.2–1.5 was generally suitable. Thus, all degradation rates in Figure 7 were suitable for biological application. While the DR in the ZX-alloy remained below 0.5 mm/year, the Zr content in the ZXK-alloy elevated the DR (Figure 7a) especially with increasing extrusion temperature and speed. Even though there was a significant variation in grain size over the parameters and with Zr content, the grain size did not correlate directly with the measured trends in DR. The only statistical differences were measured between ZXK000 5 mm/s at 300 °C and ZX00 extruded at 1 and 5 mm/s at 300 °C.

No visible variation of the DR with an average of 0.42 (0.07) mm/year was measured with the content of Ce or the change of the processing parameters (Figure 7b). It is worthwhile repeating that these materials exhibited average grain sizes varying between 5 and 19 µm, a content from 0.3 to 1.2 wt.% Ce, as well as the corresponding increasing amount of stringer particles distributed in the microstructure. Although these changes were distinct, there was no impact on the degradation behavior of this alloy series (no statistical differences).

The measured DR with the silver content (Figure 7c) were the highest and most heterogeneous measured in this study. They almost all remained in a range between 0.6 and 1.2 mm/y. An increase in the silver content seemed to reduce the DR especially at 300 °C and 1 mm/s. With this setup, a significantly low temperature was measured at the die, shown in Table 2. The addition of Zr did not show an obvious effect here. On the other hand, the grain size was almost half the size of the grains without Zr content, ca. 10 µm. Only statistical differences were measured between QXK200 (1 mm/s at 300 °C) and QXK100 (1 mm/s and 3 mm/s at 300 °C), as well as with QX20 1 mm/s at 300 °C.

**Figure 8:**
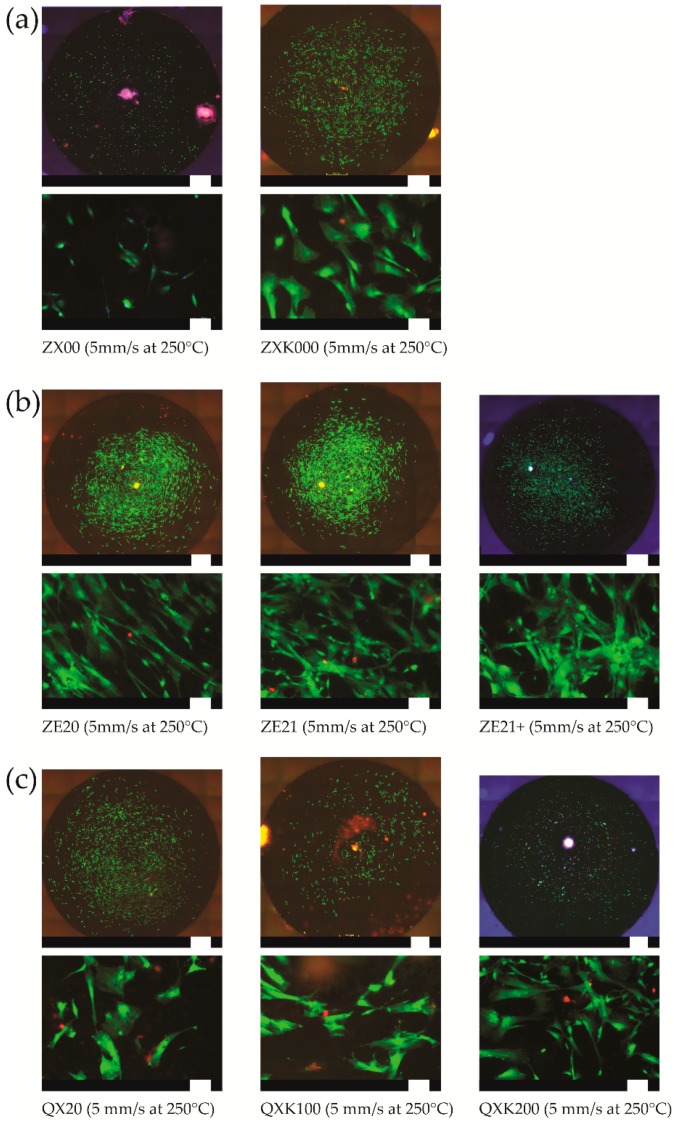
Live/dead staining of human umbilical cord perivascular cells (HUCPV) cultured on the different materials. (**a**) ZX-, (**b**) ZE-, and (**c**) QX-series. For each material, an overview micrograph (scale bar 1 mm) and a representative view with higher magnification (below the overview; scale bar 100 µm) are presented.

As no huge differences were measured in the DR, only samples with the faster extrusion speed (i.e., 5 mm/s) at 250 °C were selected for viability tests (Figure 8). As expected from the material compositions and their degradation rates, all materials possessed a rather good cytocompatibility. Cells, mostly alive, were spread on all material surfaces and exhibited a flat, fibroblastic phenotype. Very few dead, red cells could be found. For the same parameters (i.e., 5 mm/s), for the ZX-series, the addition of Zr slightly decreased the DR (0.5 ± 0.3 mm/year vs. 0.4 ± 0.3 mm/year), but also less inclusion could be seen on the sample surface (Figure 1); both observations may explain the slightly decreased cell viability on the ZX00 sample compared to the ZXK000 sample. The cell viability, as the DR, remained rather similar on all materials of the ZE series. For the QX series, variable DR were observed, increasing from QX20 (0.6 ± 0.2 mm/year), QXK100 (1.0 ± 0.4 mm/year), to QXK200 (1.0 ± 0.4 mm/year), according to decreased cell viability.

## 4. Discussion

### 4.1. Processing Effects and Microstructure Development during Extrusion

**Figure 9:**
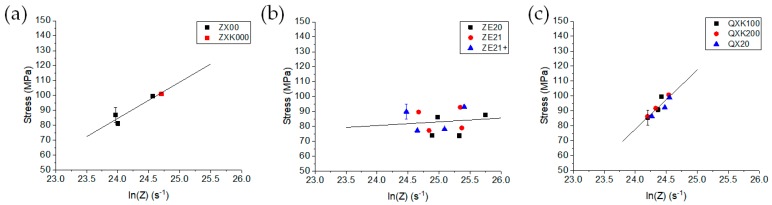
Steady state extrusion stress vs. a temperature compensated strain rate Z for the three alloy series; lines from linear regression are to guide the eyes: (**a**) ZX-series alloys, (**b**) ZE-series alloys, and (**c**) QX-series alloys.

Typical features of the microstructure development of magnesium alloys during extrusion are explained with the impact of dynamic recrystallization resulting from processing [24,39,40,41], which is largely alloy dependent. As such, the increase of the extrusion speed was associated with an enhancement of recrystallization, which can be explained by the increase of the forming temperature resulting from deformation heating due to the high degrees of applied deformation. Table 2 confirms this behavior for all magnesium alloys of this study, and the heating can be significant. In steady state flow, this can obviously result in a lower required flow stress during extrusion, which was especially visible for the Ca containing alloys. For a comprehensive assessment of these results, it is recognized that the flow stress of an alloy in a hot working scheme depends on temperature T and strain rate $\dot{\varepsilon }$. Their influence on the flow stress can be represented by a temperature compensated strain rate *Z* as suggested by Zener and Hollomon [42],

$$Z=\dot{\varepsilon }\cdot \exp\left(Q/RT\right)$$

where *Z* is labelled the “Zener–Hollomon parameter”. *Q* describes the effective activation energy of deformation, a widely unspecific and unknown property, often filled with the activation energy of self-diffusion of magnesium (135 kJ/mol) [43,44]. A relation of the steady state extrusion stress $\sigma_{extrusion}$ to the extrusion force *F* during indirect extrusion, as well as of the strain rate to the ram speed *v_ram_* can be established by:

$$\sigma_{\mathrm{extrusion}}=F/\left(C\cdot \ln\left(r\right)\cdot A\right),\;\;\;\;\;\;\;\;\;\dot{\varepsilon }\;=\;9.6\cdot r^{0.6}\cdot v_{\mathrm{ram}}/D_{\mathrm{billet}}$$

where *r* is the extrusion ratio, *A* the cross-section area of the container, *D*_billet_ the billet diameter, and *C* a specific shape efficiency factor, which was assumed to be 1.66 for a round bar extrusion [45]. Then, the results from Table 2 could be compared in the form of σ(ln(*Z*))-plots, therefore including both extrusion rate and temperature effects on the resulting extrusion stress (or the extrusion force, respectively). In Figure 9, the three alloy series are compared. For the ZX-series alloys, the increase of the stress with Z was continuous, confirming the above mentioned effect of the decrease of the extrusion force with increasing speed. Due to the lack of temperature measurements for ZXK000, a potentially different behavior of the Zr containing alloy could not be revealed. The data scatter for the results of the ZE-series alloys was much higher, but no obvious variation with the varied Ce content was found within this constraint. The strong scatter of data could be explained by the original extrusion forces in Table 2, which remained rather constant with the extrusion speed for this alloy series. The slope of the linear regression in Figure 9b was low, indicating a rather low impact of temperature and/or strain rate on the extrusion stress in comparison to the two other alloy series. In Figure 9c, however, the slope was highest for the Ag containing alloys. Again, no high variation could be found for the different alloy modifications, i.e., the change of the Ag content and the addition of Zr did not affect the hot working behavior of the alloys.

For the Ca containing alloys, Ag as an additional alloying element led to the highest temperature and strain rate dependence compared to the alloying with Zn. The Ce containing alloy series was mostly stable in its flow stress with varied temperature and strain rate. Note that for this alloy series, stable extrusion at low temperature and speed was feasible, indicating that the low stress variations were accompanied by a broader processing window.

**Figure 10:**
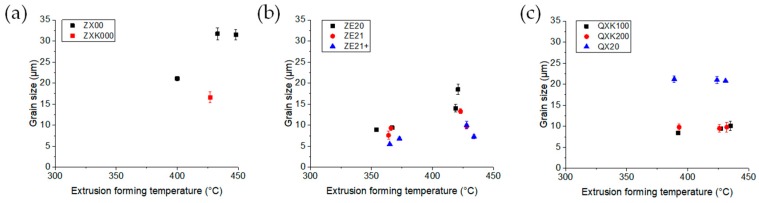
Average grain size correlation to the extrusion forming temperature (measured in the die): (**a**) ZX-series alloys, (**b**) ZE-series alloys, and (**c**) QX-series alloys.

The microstructure development during extrusion would then be primarily dependent on the forming temperature. Figure 10 enables a comparison of the temperature impact on the microstructure development, where the average grain size is plotted vs. the extrusion temperature as measured at the die during processing. A certain increase of the grain size for ZX00 appeared, resulting in rather coarse grained microstructures, obviously in a stable setting range of the extrusion parameters. Zr in this alloy (ZXK000) led to a visible refinement of the grain structure. Although extrusion temperatures for two experiments were not available, the results in Figure 1 suggested a somewhat similar tendency at lower average grain size. In the same range of extrusion parameters and a resulting similar temperature range for the Ag containing alloys, no such clear variation was revealed despite the distinct impact of the extrusion parameters on the extrusion flow stress. Again, Zr acted as a grain refiner during recrystallization. A similar stabilization of the grain structure was seen for the ZE-series alloys where the increase of the average grain size with temperature slowed down as the content of Ce increased, leading to the finest grained microstructures of this study.

### 4.2. Influence of the Alloy Composition on the Microstructure Development

The most distinct impact of rare earth elements or Ca as alloying elements to magnesium was the refinement of the microstructures and a texture change, which allowed the development of a texture component with tilt basal planes [24,41]. All alloys containing Ca in this study exhibited such a tilt component of the basal planes out of the extrusion direction. If earlier works concentrated on the behavior of binary Mg-Ca alloys during extrusion, it could be concluded that Ag and Zn as additional alloying elements in the ZX-series and QX-series alloys (with their rather limited contents) did not change this microstructure and texture development in the first place [46,47]. Completely recrystallized microstructures were formed.

Although the ZE-series alloys would also be likely to develop such a rare earth texture component [38,40], the results in this study revealed comparably weak textures where the preferred orientation of the basal planes along the extrusion direction was maintained and concurrent to the very fine grained microstructures. Although no partial recrystallization could be detected in these microstructures with increasing Ce content, the intensity at the <10$\bar{1}$0> pole increased visibly. While this component has often been assigned to the non-recrystallized fraction of the microstructure [48], it could also be found if the recrystallization process from these microstructure fractions took place more slowly [24,49]. This behavior was apparently intensified by the addition of Ce, although initially, only an increase in the content of intermetallic particles could be observed in this alloy. In this case, a strong <10$\bar{1}$0> fiber with its concurrent alignment of basal planes parallel to ED was maintained during recrystallization, and only a rotation around the c-axis was associated with the recrystallization mechanisms [50]. It was therefore hypothesized that the retarded onset of (dynamic) recrystallization led to maintaining an alignment of basal planes parallel to ED, whereas in cases with enhanced dynamic recrystallization, a tilt texture component was more stabilized, as observed in the case of the Ca containing alloys. While this behavior would be associated with the nucleation of grains, a retardation of grain boundary mobility was associated with the stabilization of the rare earth textures [51], but not with stable orientations during the nucleation of recrystallized grains.

Interestingly, in the case of the QX alloy, the grain structure was independent of the process parameters within the process window used, i.e., the microstructure development was stabilized against the obvious changes in the flow behavior of the material. However, the extrusion of these alloys was carried out in a temperature and alloying range where stable precipitates were revealed (like in the ZE-series alloys). In the case of QX20, without Zr, the above mentioned stable grain sizes were higher compared to the ZE-series alloys, although not varying. The presence of the respective precipitates, which of course varied in nature, were consistent with a retardation of grain growth, not with a retardation of grain nucleation. Then, Ca did not retard the nucleation of recrystallized grains during extrusion as Ce did, and the rare earth texture component was developed.

Unlike this, in ZX00, there was a clear dependency of the grain size on the deformation temperature and a much more coarsely grained microstructure. In only this alloy, where solid solution annealing could be applied, the principle absence of precipitates led to the formation of such microstructures within the corresponding process window. Still, the texture of this Ca containing alloy maintained the character of tilted basal planes.

In all alloys with Zr, a grain refinement could be observed compared to the respective Zr free counterpart alloys. A decrease in grain size was still weakly pronounced at a content of 0.1% in ZXK000, while a higher content of 0.3% in the QX alloys compared to the alloy without Zr resulted in a significantly more pronounced grain refinement. The influence of Zr as a grain finer in the casting process has been known. In the case of thermomechanical treatment during extrusion, the addition of Zr could be correlated with a retardation of grain growth [52], but not in principle with a change in microstructure development, since no changes in texture development could be detected.

### 4.3. Alloying Impact on the Mechanical Behavior

**Figure 11:**
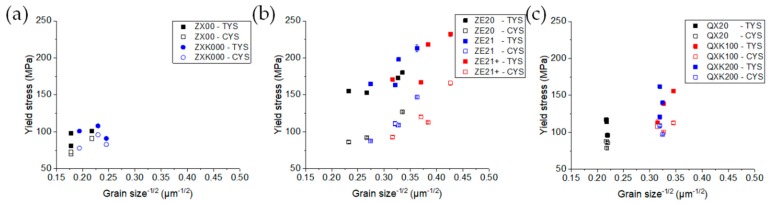
Yield stresses in tension and compression vs. the inverse square root of the grain size (Hall–Petch plot): (**a**) ZX-series alloys, (**b**) ZE-series alloys, and (**c**) QX-series alloys.

For the ZE-series alloys, the yield strength could be reproduced by mainly considering a direction specific grain size strengthening behavior; see Figure 11, where the relation between the TYS or CYS vs. the inverse square root of the average grain size, following $YS\sim 1/\sqrt{grain\;size}$, was shown (Hall–Petch plots). The lower yield stress during compressive loading and also the subsequent S shape of the stress–strain curves (Figure 6) were associated with a preferred activation of twins, which resulted from the texture related compression axis perpendicular to the c-axis of the lattice and thus a resulting tensile strain parallel to the c-axis [53]. Within the rather large scatter in Figure 11b, no clear difference in the grain size related strengthening between tension and compression could be revealed. However, the increase of the Ce content shifted the analyzed alloys towards more finely grained microstructures and correspondingly higher yield stresses. Even if the increase of the Ce content in the form of solute or precipitates allowed assuming an element related strengthening effect, it was not resolved over the grain size related strengthening of the alloys. Furthermore, the scatter of the respective yield stresses with the grain size was also based on differences in the texture of the samples: the weakest textures led to the lowest yield stresses for the three alloys. In this case, the alignment of the basal planes along the extrusion direction was less pronounced, which correlated with an increased activation ability of basal slip and a corresponding decrease in the yield stress. It is noteworthy that the finest grain structures with the highest stress values were extruded at low speed at the higher temperature (300 °C).

A similar plot for the ZX alloys (Figure 11a) or the QX-series alloys (Figure 11c) was less clear, although the respective grain size strengthening was also visible. However, those samples with the most pronounced textures (opposite the weaker textures for the ZE-series alloys), i.e., with the most pronounced tilt component of the basal planes, now led to the lowest yield stresses for both alloys ZX00 and ZXK000, each the finest grained samples. Again, the Zr containing alloys with their more finely grained microstructures compared to ZX00 did not exhibit a specific impact of Zr on the stress levels reached during yielding. Note that in these cases, the yield asymmetry between tension and compression was lowest. For the QX-alloys, samples that showed the slight development of a <10$\bar{1}$0> component exhibited an increased yield asymmetry. In this alloy series, the Zr related grain refinement increased the yield stress levels, but the strong impact of small texture changes, varying between the above described effects for the alignment of basal planes like for the ZE-series alloys and the tilt component like in the ZX-series alloys, had an over-ruling impact on the mechanical properties.

### 4.4. Alloy Specific Degradation Behavior and Cytocompatibility

All alloy series exhibited suitable degradation rates for biological applications. Degradation rates within and between the series remained rather constant independent of the material properties (e.g., extrusion speed and temperature) and the material compositions, offering a wide range of possibility or choice.

The lowest and rather homogeneous degradation behavior was found for ZX00. Zr in this alloy led to a visible increase and a more heterogeneous degradation behavior. For the ZE series, here also, a rather homogenous degradation was found independent of the composition and the processing parameters, all leading to a good cytocompatibility. In the QX-series alloys, there was no distinct dependency on the Zr content, but the initial degradation rates in the QX20 alloy were already higher. The concurrent increase of degradation as a result of Ag containing particles was consistent with earlier work [22], where Ag in solid solution allowed maintaining lower rates and more homogeneous degradation, but conditions with such precipitates resulted in an increased rate. Again, this confirmed the impact of particles with different electrochemical potential. There was no such impact from the precipitates in the ZE-series alloys.

## 5. Conclusions

The three different alloy series of this study, Mg-Zn-Ca(Zr), Mg-Zn-Ce, and Mg-Ag-Ca(Zr), allowed the comparison of the processing effects during extrusion, the resulting microstructure and texture development, and the related mechanical properties. While in these dilute alloys, single effects of the alloying elements on the mechanical properties were not revealed, their impact resulted from effects on the extrusion processing windows and the corresponding microstructure and texture development. The resulting mechanical properties were very consistent with grain size and texture effects, only. Finely grained microstructures led to high yield stresses, whereas textures with a tilt of the basal planes enhanced strain properties in tensile and compression testing at the expense of strength.

The ZE-series alloys with Ce as the alloying element resulted in broad and stable extrusion process windows, whereas for all Ca containing alloys, extrusion at lower speed and temperature was not stable.

An expected impact on the microstructure refinement was therefore limited. However, Zr acted as a grain refiner in the recrystallization dominated microstructure development during extrusion. Particle stabilization of grain growth during recrystallization like in the ZE-series alloys and the QX-series alloys was distinct, whereas solid solution, approximately, in ZX00 did not have such a grain growth limiting effect.

A retardation of grain nucleation vs. grain growth could be hypothesized in the Ce containing alloys, therefore maintaining deformation related alignment of basal planes during recrystallization. Unlike this, all Ca containing alloys developed a tilt component of basal planes with enhanced tendency to grain nucleation.

While degradation rates were variable for the different alloy series, they all remained in a range below 1.5 mm/y, thus proving suitable conditions for biological applications. Variation due to grain size changes was not found to have a significant impact, and Zr and Ag containing alloys revealed higher rates and more distinct degradation inhomogeneity. Still, all alloys exhibited promising cytocompatibility.

This study showed the interconnections and degrees of freedom for the design of implants with tailored microstructure and mechanical properties that were achievable while maintaining excellent cell compatibility.

## Figures and Tables

**Table 1:** Alloy labels and chemical compositions of the cast billets including main alloying elements and main impurity elements in wt.%, Mg balance.

Alloy Label	Zn	Ce	Ca	Ag	Zr	Fe	Cu	Ni
ZX00	0.49	–	0.35	–	–	0.003	0.002	0.002
ZXK000	0.52	–	0.24	–	0.06	0.003	0.002	0.002
ZE20	2.10	0.27	–	–	–	0.002	0.003	<0.002
ZE21	1.96	0.72	–	–	–	0.003	0.002	<0.002
ZE21+	1.97	1.15	–	–	–	0.003	0.002	<0.002
QX20	–	–	0.41	2.00	–	0.034	0.001	0.002
QXK100	–	–	0.42	1.02	0.31	0.004	0.001	0.002
QXK200	–	–	0.41	1.94	0.27	0.004	0.001	0.001

**Table 2:** Steady state forces and steady state extrusion temperatures for the alloys of this study during extrusion with varied temperatures and extrusion speeds; accuracy for the force ±0.2 MN; accuracy for the temperature measurement ±2 °C; n/a: these data were not accessible during the measurements; unstable: no steady state flow reached.

Alloy	Steady State Force (MN)				Extrusion Temperature at the Die (°C)			
	Extrusion at 250 °C 1 mm/s	Extrusion at 250 °C 5 mm/s	Extrusion at 300 °C 1 mm/s	Extrusion at 300 °C 5 mm/s *	Extrusion at 250 °C 1 mm/s	Extrusion at 250 °C 5 mm/s	Extrusion at 300 °C 1 mm/s	Extrusion at 300 °C 5 mm/s *
ZX00	unstable	5.2	3.6	3.3	unstable	425	400	447
ZXK000	unstable	4.8	3.6	3.3	unstable	430	n/a	n/a
ZE20	3.5	3.5	3.0	3.0	355	418	368	422
ZE21	3.7	3.6	3.2	3.1	364	427	368	423
ZE21+	3.8	3.6	3.2	3.1	366	432	377	429
QX20	unstable	4.0	3.8	3.5 *	unstable	430	388	424 *
QXK100	unstable	4.0	3.7	3.5 *	unstable	433	393	428 *
QXK200	unstable	4.0	3.7	3.5 *	unstable	430	392	427 *

* 3 mm/s for QX(K) series alloys.

**Table 3:** Mechanical properties from tensile and compression tests in Figure 2a,b for ZX00 and ZXK000; TYS: tensile yield stress, UTS: ultimate tensile stress, CYS: compressive yield stress, UCS: ultimate compressive stress; errors in brackets.

Alloy	Condition	TYS (MPa)	UTS (MPa)	Uniform Strain (%)	Fracture Strain (Tension) (%)	CYS (MPa)	UCS (MPa)	Fracture Strain (Comp.) (%)
ZX00	5 mm/s, 250 °C	81 (2)	201 (1)	23.0 (0.4)	35.3 (0.9)	70 (1)	327 (7)	23.4 (2.0)
	1 mm/s, 300 °C	101 (1)	209 (1)	19.7 (0.5)	32.8 (1.2)	91 (1)	323 (2)	17.7 (1.4)
	5 mm/s, 300 °C	98 (2)	205 (1)	19.7 (0.6)	29.1 (0.5)	73 (1)	311 (3)	18.5 (0.7)
ZXK000	5 mm/s, 250 °C	91 (1)	207 (1)	21.1 (0.3)	35.5 (1.4)	83 (1)	317 (3)	18.3 (1.6)
	1 mm/s, 300 °C	108 (1)	212 (1)	19.0 (0.6)	32.2 (0.9)	96 (1)	323 (2)	16.4 (0.7)
	5 mm/s, 300 °C	101 (2)	209 (1)	18.3 (0.3)	29.5 (0.3)	78 (1)	312 (2)	16.6 (1.5)

**Table 4:** Mechanical properties from tensile and compression tests in Figure 4 for the ZE-series alloys TYS: tensile yield stress, UTS: ultimate tensile stress, CYS: compressive yield stress, UCS: ultimate compressive stress; errors in brackets.

Alloy	Condition	TYS (MPa)	UTS (MPa)	Uniform Strain (%)	Fracture Strain (Tension) (%)	CYS (MPa)	UCS (MPa)	Fracture Strain (Comp.) (%)
ZE20	1 mm/s, 250 °C	180 (1)	254 (1)	13.0 (0.4)	26.4 (1.8)	127 (1)	392 (7)	12.0 (1.0)
	5 mm/s, 250 °C	153 (2)	235 (1)	12.0 (0.2)	25.0 (0.8)	92 (1)	350 (2)	11.7 (1.0)
	1 mm/s, 300 °C	173 (1)	248 (1)	11.5 (0.3)	24.4 (0.6)	109 (1)	380 (5)	10.9 (0.3)
	5 mm/s, 300 °C	155 (1)	234 (1)	11.1 (0.2)	25.2 (1.5)	86 (2)	355 (2)	11.3 (0.3)
ZE21	1 mm/s, 250 °C	213 (5)	264 (3)	13.2 (0.2)	29.1 (3.1)	147 (1)	411 (4)	10.6 (0.8)
	5 mm/s, 250 °C	163 (1)	239 (1)	12.3 (0.1)	27.1 (2.3)	111 (2)	361 (6)	11.3 (0.9)
	1 mm/s, 300 °C	198 (2)	257 (1)	11.9 (0.2)	27.6 (1.3)	109 (1)	337 (3)	10.3 (0.4)
	5 mm/s, 300 °C	165 (1)	239 (1)	11.3 (0.2)	27.2 (0.8)	88 (1)	302 (1)	10.2 (0.3)
ZE21+	1 mm/s, 250 °C	232 (3)	272 (2)	12.2 (0.3)	26.0 (1.6)	166 (3)	420 (9)	9.9 (0.6)
	5 mm/s, 250 °C	167 (1)	238 (1)	12.3 (0.2)	26.8 (1.5)	120 (2)	367 (4)	10.9 (0.5)
	1 mm/s, 300 °C	218 (2)	265 (1)	10.9 (0.3)	24.9 (1.2)	113 (1)	347 (3)	9.7 (0.3)
	5 mm/s, 300 °C	171 (1)	239 (1)	10.7 (0.2)	27.0 (2.0)	93 (2)	302 (4)	10.0 (0.4)

**Table 5:** Mechanical properties from tensile and compression tests in Figure 6 for the QX-series alloys. TYS: tensile yield stress, UTS: ultimate tensile stress, CYS: compressive yield stress, UCS: ultimate compressive stress; errors in brackets.

Alloy	Condition	TYS (MPa)	UTS (MPa)	Uniform Strain (%)	Fracture Strain (Tension) (%)	CYS (MPa)	UCS (MPa)	Fracture Strain (Comp.) (%)
QX20	5 mm/s, 250 °C	96 (3)	220 (1)	24.2 (0.5)	33.1 (1.1)	86 (2)	337 (9)	20.4 (1.3)
	1 mm/s, 300 °C	117 (1)	227 (1)	21.9 (0.5)	33.9 (0.9)	88 (1)	289 (4)	15.6 (0.5)
	3 mm/s, 300 °C	114 (1)	226 (1)	21.1 (0.5)	29.8 (1.1)	79 (1)	291 (5)	17.0 (1.5)
QXK100	5 mm/s, 250 °C	121 (3)	218 (1)	19.5 (1.0)	27.1 (6.5)	109 (1)	313 (2)	15.2 (0.4)
	1 mm/s, 300 °C	162 (2)	233 (1)	15.9 (0.3)	30.2 (1.1)	109 (3)	291 (3)	11.5 (0.5)
	3 mm/s, 300 °C	140 (2)	224 (1)	17.1 (0.4)	27.6 (2.4)	97 (1)	279 (2)	12.1 (1.0)
QXK200	5 mm/s, 250 °C	113 (4)	228 (1)	22.4 (0.3)	27.4 (4.7)	108 (3)	322 (6)	14.8 (1.2)
	1 mm/s, 300 °C	156 (1)	242 (1)	18.3 (0.2)	31.5 (1.8)	113 (2)	295 (5)	12.0 (0.3)
	3 mm/s, 300 °C	139 (1)	234 (1)	18.8 (0.2)	28.2 (1.9)	100 (1)	279 (6)	11.8 (0.7)
